# Antibacterial Effect of Dental Adhesive Containing Dimethylaminododecyl Methacrylate on the Development of *Streptococcus mutans* Biofilm

**DOI:** 10.3390/ijms150712791

**Published:** 2014-07-18

**Authors:** Suping Wang, Keke Zhang, Xuedong Zhou, Ning Xu, Hockin H. K. Xu, Michael D. Weir, Yang Ge, Shida Wang, Mingyun Li, Yuqing Li, Xin Xu, Lei Cheng

**Affiliations:** 1 State Key Laboratory of Oral Diseases, Sichuan University, Wuhou District, Chengdu 610041, China; E-Mails: wangsupingdent@163.com (S.W.); zalmancoco@163.com (K.Z.); zhouxd@scu.edu.cn (X.Z.); xuning880804@126.com (N.X.); yamy111@163.com (Y.G.); wangshida1987@gmail.com (S.W.); limingyun@scu.edu.cn (M.L.); liyuqing13@163.com (Y.L.); nixux1982@hotmail.com (X.X.); 2 Department of Operative Dentistry and Endodontics, West China Hospital of Stomatology, Sichuan University, Chengdu 610041, China; 3 Biomaterials & Tissue Engineering Division, Department of Endodontics, Prosthodontics and Operative Dentistry, University of Maryland Dental School, Baltimore, MD 21201, USA; E-Mails: Hxu@umaryland.edu (H.H.K.X.); MWeir@umaryland.edu (M.D.W.)

**Keywords:** antibacterial adhesive, DMADDM, *S. mutans* biofilm, microtensile

## Abstract

Antibacterial bonding agents and composites containing dimethylaminododecyl methacrylate (DMADDM) have been recently developed. The objectives of this study were to investigate the antibacterial effect of novel adhesives containing different mass fractions of DMADDM on *Streptococcus mutans* (*S. mutans*) biofilm at different developmental stages. Different mass fractions of DMADDM were incorporated into adhesives and *S. mutans* biofilm at different developmetal stages were analyzed by MTT assays, lactic acid measurement, confocal laser scanning microscopy and scanning electron microscopy observations. Exopolysaccharides (EPS) staining was used to analyze the inhibitory effect of DMADDM on the biofilm extracellular matrix. Dentin microtensile strengths were also measured. Cured adhesives containing DMADDM could greatly reduce metabolic activity and lactic acid production during the development of *S. mutans* biofilms (*p* < 0.05). In earlier stages of biofilm development, there were no significant differences of inhibitory effects between the 2.5% DMADDM and 5% DMADDM group. However, after 72 h, the anti-biofilm effects of adhesives containing 5% DMADDM were significantly stronger than any other group. Incorporation of DMADDM into adhesive did not adversely affect dentin bond strength. In conclusion, adhesives containing DMADDM inhibited the growth, lactic acid production and EPS metabolism of *S. mutans* biofilm at different stages, with no adverse effect on its dentin adhesive bond strength. The bonding agents have the potential to control dental biofilms and combat tooth decay, and DMADDM is promising for use in a wide range of dental adhesive systems and restoratives.

## 1. Introduction

Dental caries are considered one of the most prevalent chronic diseases of people worldwide [1]. Caries are still a heavy burden around the world though the disease process and the potential to prevent and control the disease are now much better understood [1,2]. Nowadays, resin composites are becoming popular filling materials due to their esthetics and direct-filling capabilities in the treatment of dental caries [3,4,5,6,7,8,9,10]. However, half of all restorations fail within 10 years, and replacing them consumes 50% to 70% of the dentists’ time [11]. Replacement dentistry costs $5 billion annually in the U.S [12]. Previous investigation indicated that secondary caries is still a great challenge for the dental restoration [7].

Composite restorations are bonded to the tooth structure via adhesives system [13,14]. Previous studies have enhanced the bond strength and elucidated the mechanisms of adhesion [14,15,16,17,18]. It is beneficial for the adhesive to be able to reduce biofilm acids and secondary caries [19,20]. Besides residual bacteria in the prepared tooth cavity, bacteria might invade through marginal leakage and cause secondary caries. Therefore, antibacterial adhesives are being developed to help inhibit the residual as well as invading bacteria [21,22]. Varied methods are used to make adhesives antibacterial. Some are based on materials that contain antibacterial agents or groups, while some studies show that adhesives can be made antibacterial on-demand without introducing antibacterial entities in the material by photocatalysis [23]. Quaternary ammonium salts (QAS) are one group of antibacterial agents [24,25]. Antibacterial QAS monomers copolymerize with other monomers in the composite and adhesives to form polymer matrices that can combat bacteria, such as 12-methacryloyloxydodecylpyridinium bromide (MDPB) [20,21,22,25], quaternary ammonium dimethacrylate (QADM) [26,27,28], methacryloxyl ethyl cetyl dimethyl ammonium chloride (DMAE-CB) [29] and so on. In previous studies, a new quaternary ammonium monomer, dimethylaminododecyl methacrylate (DMADDM), was synthesized and proved to be much more strongly antibacterial than QADM. Meanwhile, DMADDM in a fourth generation adhesive system was found to have strong antibacterial effect on the mature microcosm biofilms [30]. However, there are still some questions about the new antibacterial monomers. Firstly, the antibacterial mechanism of QAS is “contact-killing” [5,8], so it is interesting to investigate how the antibacterial monomer influences biofilms at different developing stages, from early adhesion to mature biofilms. Secondly, all the previous studies focused on the effect of DMADDM on the bacteria in biofilm [30]; no previous paper investigated the effect of DMADDM on the biofilm matrix. Matrix constituents, such as exopolysaccharides (EPS), could affect the diffusion of substances in and out of the biofilm, perhaps helping to create a diverse range of microenvironments within the biofilm [31].

The objectives of this study were to incorporate the new DMADDM into adhesives, and to investigate the effects on *S.* *mutans* biofilms and dentin bonding properties. It was hypothesized that: (1) Adhesive containing DMADDM will inhibit *S. mutans* biofilm at different stages; (2) DMADDM in adhesives will inhibit both the bacteria and EPS production in biofilm.

## 2. Results

### 2.1. Microtensile Bond Strength Test

**Figure 1:**
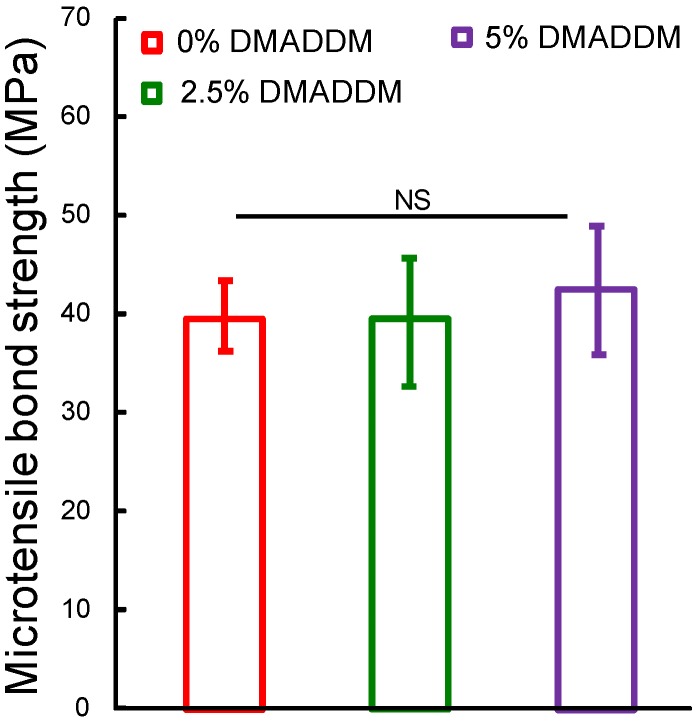
Microtensile strength of adhesive containing different mass fractions of DMADDM. Each values is mean ± SD (*n* = 10) (*p* > 0.1).

The microtensile strength results are illustrated in Figure 1 (mean ± SD; *n* = 10). The three groups had microtensile strengths that were not significantly different from each other (*p* > 0.1). In the tested mass fraction, the results demonstrate that incorporation of DMADDM into adhesives did not adversely affect the dentin microtensile strengths.

### 2.2. MTT Assays

**Figure 2:**
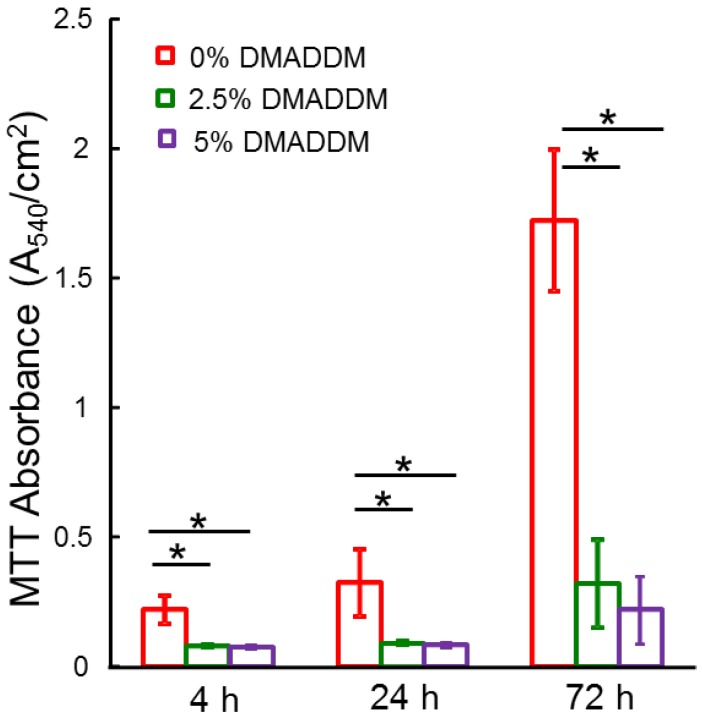
MTT metabolic activity in three groups. Biofilms were grown for 4, 24 and 72 h days using an *S.* *mutans* model. Each values is mean ± SD (*n* = 6) (* *p* < 0.05).

The biofilm metabolic activity was measured using the MTT assay and the results were plotted in Figure 2. There was a monotonic decrease in MTT with increasing DMADDM content. After 4, 24 and 72 h growth, the MTT results (mean ± SD; *n* = 6) in Figure 2 shows that biofilms in the control group had a high metabolic activity compared to the other two groups. At all the time points, incorporation of 2.5% DMADDM into the adhesive greatly reduced the metabolic activity compared to the control group (* *p* < 0.05 ). In the group incorporating 5% DMADDM in the adhesive, the lowest metabolic activity was achieved compared to other two groups. Adhesive containing 5% DMADDM yielded a biofilm metabolic activity that was at least 2-fold lower than that of commercial bonding agent control.

### 2.3. Lactic Acid Measurement

**Figure 3:**
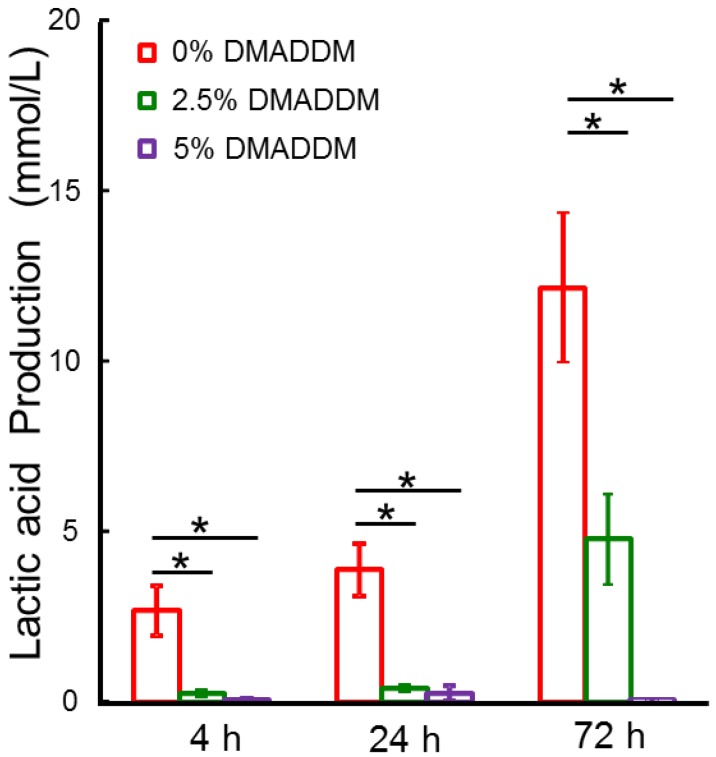
Lactic acid production by *S. mutans* biofilms adherent on the disks. Each value is mean ± SD; *n* = 6 (* *p* < 0.05).

The lactic acid production by biofilms is plotted in Figure 3 (mean ± SD; *n* = 6). Biofilms in commercial control group produced the most lactic acid at different developing stages. Adding 2.5% of DMADDM in adhesive greatly reduced acid production, compared to the control (* *p* < 0.05). Using 5% DMADDM had a significantly stronger acid-inhibiting effect than using 2.5% DMADDM after 72 h growth (* *p* < 0.05). Lactic acid production by biofilms on 5% DMADDM was approximately 1/10 of that on the commercial bonding agent after 4 h growth, but 1/50 after 72 h growth.

### 2.4. Live/Dead Bacteria Staining

**Figure 4:**
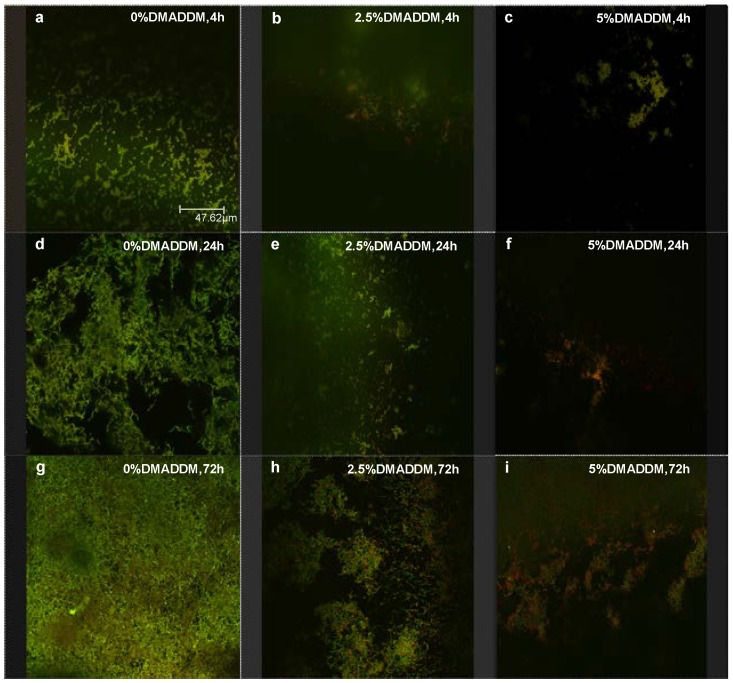
Live/dead bacterial staining of biofilms on the cured disks of the three groups. Each group name is indicated in the image. Live bacteria were stained green, and dead bacteria were stained red. Live and dead bacteria in close proximity to each other yielded yellow and orange colors. Incorporation of DMADDM into the adhesive provided strong antibacterial effects. (**a**) 0% DMADDM group, 4 h; (**b**)2.5% DMADDM group, 4 h; (**c**) 5%DMADDM group, 4 h; (**d**) 0% DMADDM group, 24h; (**e**) 2.5% DMADDM group, 24h ; (**f**) 5% DMADDM group, 24 h; (**g**) 0% DMADDM group, 72 h; (**h**) 2.5%DMADDM group, 72 h; (**i**) 5% DMADDM group, 72 h.

Figure 4 shows typical live/dead staining confocal images of biofilms on adhesives at 4, 24 and 72 h. Live bacteria were stained green, and the dead bacteria were stained red. Biofilms on control disks became obviously denser from 4 to 72 h. The images show primarily live bacteria, with small amounts of dead cells (Figure 4a,d,g). In contrast, substantial increases in dead bacteria occurred when adhesive contained 2.5% DMADDM (Figure 4b,e,h). When 5% DMADDM was added into adhesive, there were much less bacteria on the disk and the biofilms consisted of primarily dead bacteria (Figure 4c,f,i).

### 2.5. Exopolysaccharides (EPS) Staining

**Figure 5:**
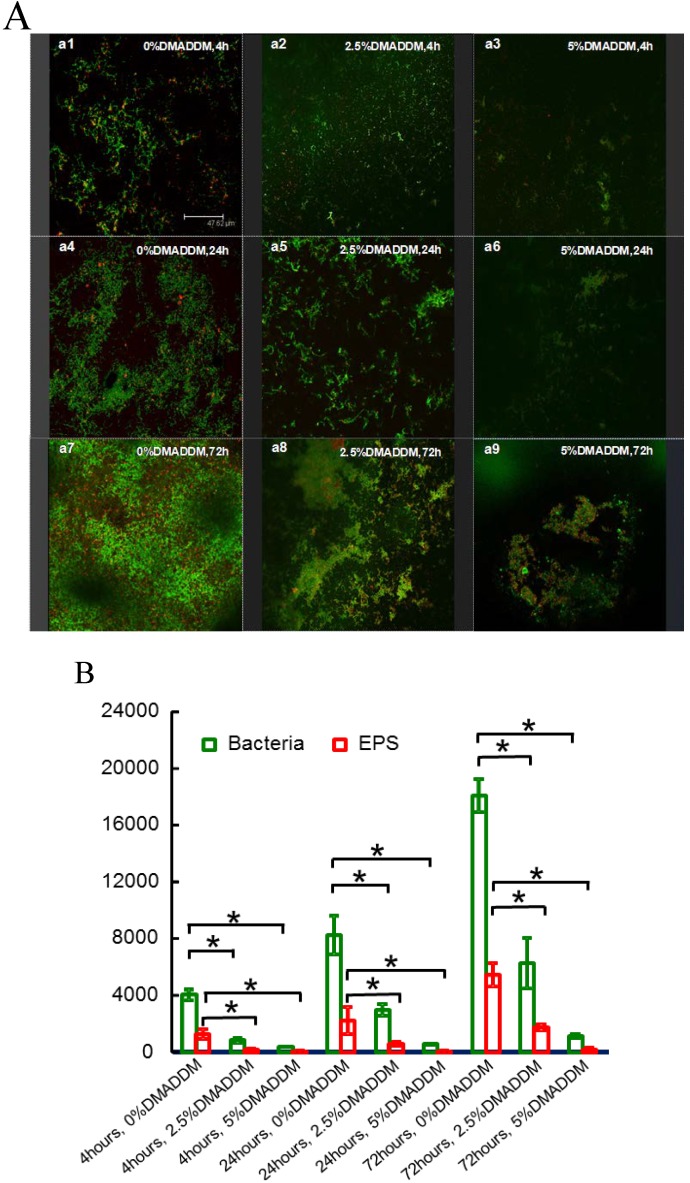
(**A**) EPS staining of biofilms on the cured disks of the three groups at different stages. Bacteria were stained green, and EPS were stained red. Incorporation of DMADDM into the adhesive reduced both the bacteria and EPS; (**B**) Part quantified analysis of EPS production and bacteria. Results were averaged from three randomly selected views of each group and are presented as mean ± standard deviation (* *p* < 0.05). (**a1**) 0% DMADDM group, 4 h; (**a2**) 2.5% DMADDM group, 4 h; (**a3**) 5% DMADDM group, 4 h; (**a4**) 0%DMADDM group, 24 h; (**a5**) 2.5% DMADDM group, 24 h; (**a6**) 5% DMADDM group, 24 h; (**a7**) 0%DMADDM group, 72 h; (**a8**) 2.5% DMADDM group, 72 h; (**a9**) 5% DMADDM group, 72 h. Scale bar = 47.62 μm.

Figure 5A shows the distribution of bacteria and EPS in the biofilms at different time points in different groups. In the control group (Figure 5A(a1,4,7)), more bacteria (green color) and EPS (red color) were observed, compared with other groups. Both the bacteria and EPS in biofilm decreased in the 2.5% DMADDM group (Figure 5A(a2,5,8)). When 5% DMADDM was added into the adhesive, biofilms had fewer bacteria and the EPS was also reduced significantly (Figure 5A(a3,6,9)). The value of relative fluorescence of EPS production and bacteria is shown in Figure 5B. With the addition of DMADDM, EPS production decreased to some extent at different growth stages, compared with the control group.

### 2.6. Scanning Electron Microscopy (SEM) Observation

**Figure 6:**
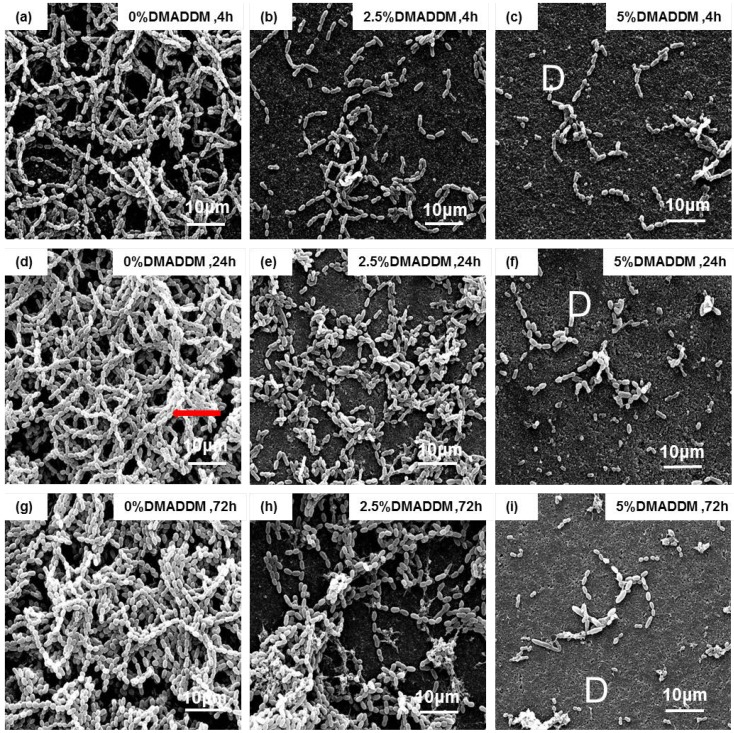
SEM micrographs of typical biofilms in different groups. Each group name is indicated in the image. Adhesives containing DMADDM could reduce the biofilm growing on the disks. Higher mass fraction of DMADDM could have higher anti-biofilm effect. (**a**) 0% DMADDM group, 4 h; (**b**) 2.5% DMADDM group, 4 h; (**c**) 5% DMADDM group, 4 h; (**d**) 0% DMADDM group, 24 h; (**e**) 2.5% DMADDM group, 24 h; (**f**) 5% DMADDM group, 24 h; (**g**) 0% DMADDM group, 72 h; (**h**) 2.5% DMADDM group, 72 h; (**i**) 5% DMADDM group, 72 h; “**D**”, disk surface without biofilms; the red line 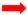 indicates *S. mutans* in chains; Scale bar = 10 μm.

The SEM images of each group at different time points are shown in Figure 6. Disks in the control group had dense biofilm, compared with the other two groups (Figure 6a,d,g). The group with 2.5% DMADDM had less bacteria in the biofilm than the control group (Figure 6b,e,h). Even after 72 h, “D” indicating disk surface not covered by biofilms, could also be observed (Figure 6c,f,i). In the 5% DMADDM group, only a few bacteria grew on the surface of the disk (Figure 6c,f,i). The *S. mutans* grew in chains (arrows). The chains twisted in three dimensions and were long or continuous in the biofilm architecture. The chains were much shorter in the DMADDM groups.

## 3. Discussion

The present study investigated the antibacterial effect of dental adhesive containing different mass fraction of DMADDM on the *S.* *mutans* biofilm during different development stages for the first time. DMADDM with a carbon chain length of 12 had a strong inhibiting effect on the *S. mutans* biofilms. The results indicated that both lower and higher mass fractions of DMADDM could significantly decrease the growth and metabolism of *S. mutans* biofilms. Higher mass fractions of DMADDM in adhesives showed stronger inhibiting effects on the mature biofilms (72 h biofilms). Both bacteria and matrix in biofilms could be reduced by novel antibacterial bonding agents. The results of microtensile testing, as an indicator reflecting adhesive bonding strength, proved that the addition of 2.5% or 5% DMADDM into adhesive had no adverse effect on adhesive bonding strength of dental adhesive compared to the control group.

In the present experiment, a biofilm model based on 24-well plates was chosen. *S. mutans* was selected because it is a cariogenic bacterium and the primary causative agent of dental caries. *S. mutans* in dental plaque biofilms metabolize carbohydrates to acids, causing demineralization of the tooth structure and the tooth-restoration margins beneath the biofilm. Therefore this new bonding agent, which exhibits a great reduction in *S. mutans* biofilm growth and acid production, suggests a promising approach to combat recurrent caries. According to previous studies and preliminary tests, three time points were selected: 4, 24 and 72 h. During the first 4 h, bacteria in growth medium adhered to the surface of disks [32,33,34]. After 24 h, biofilms on the disks were still at the early stage of development [35,36,37]. And after 72 h culture, mature biofilms were formed on the disks [38]. In the control group, both bacteria and EPS obviously increased from the 4 h biofilms to 72 h biofilms. When DMADDM was added into adhesives, bacteria in biofilms grew slowly according to the SEM images and live-dead bacterial staining images. Even after 72 h culture, MTT assays and lactic acid measurement indicated that both bacteria and acid production declined significantly. In addition, EPS production was decreased in DMADDM groups compared with those in the control group. So the anti-biofilm effect of DMADDM lasted through the whole development process of *S. mutans* biofilms.

In a previous study, 5% DMADDM was added into the dental adhesives [30]. In the present study, lower mass fractions of DMADDM (2.5%) was added into dental adhesives and their anti-biofilm effect was investigated and compared with higher mass fraction (5%) of DMADDM. The results indicated that adhesive containing 2.5% DMADDM also showed strong antibacterial effects on the *S. mutans* biofilms. In the earlier stages, there were no significant differences in inhibiting biofilms between the 2.5% and 5% groups. But when mature biofilms were analyzed, the anti-biofilm effects of adhesives containing 5% DMADDM were significantly stronger than in any other group. Figure 3 indicated that lactic acid production in the 5% DMADDM group was less than 1 mmol/L at three time points. In the 2.5% DMADDM group, lactic acid production of 72 h biofilms was significantly higher than those of 24 h biofilms. These results indicate that 5% DMADDM group had a stronger and more inhibitory effect on the acid metabolisms of *S. mutans* mature biofilms than the 2.5% DMADDM and control groups.

Biofilms are composed of the bacteria and the extracellular matrix. Matrix constituents, such as EPS, play an important part in the development and the virulence of the biofilm. Previous studies investigated antibacterial quaternary ammonium methacrylates (QAMs) on the viability and metabolism of bacteria in biofilms. The present investigation studied the effect of DMADDM on both the bacteria and extracellular polysaccharide (EPS) in biofilms for the first time. Obviously, Figure 5 demonstrated that EPS production declined with the increase of additive amounts of DMADDM, compared to control groups at each growth stage. Considered that EPS is mainly responsible for cellular aggregation and surface attachment [39], it could be deduced that the reduced EPS production can help DMADDM exert better anti-biofilm effect. However, further research is needed to explore whether EPS related genes are also inhibited by DMADDM.

Previous studies focused on the detailed antimicrobial mechanism of QAS. It appears that QAS materials can cause bacteria lysis by binding to the cell membrane and causing cytoplasmic leakage [40]. This could happen when the negatively charged bacterial cell contacts the positively charged (N^+^) sites of the QAS resin. This disturbs the electric balance of the cell membrane, and the bacteria could lyse under its own osmotic pressure [41]; thus, QAS had a “contact-killing” effect on the bacteria. So in the earlier stages, DMADDM in the adhesives could kill the bacteria on the surface directly. MTT assays data and SEM morphology showed the strong antibacterial effect of DMADDM on the earlier *S.* *mutans* biofilms at 4 h. Biofilm development is a process influenced by the physico-chemical properties of the underlying surface. After the early bacterial adhesion to the surface, the major growth of dental plaque mass then occurs by bacterial cell division within the biofilm, rather than by co-aggregation at the surface of the developing biofilm [42]. In the present study, adhesives containing DMADDM could have significant inhibitory effects on *S. mutans* biofilms from initial to mature stages. However, the anti-biofilm mechanism of DMADDM is still unclear. It is known that DMADDM has a “contact-killing” effect on the bacteria. However, the interactions among oral bacteria are integral to the development and maturation of the biofilm, so the earlier bacterial adhesion would influence the further development of biofilms. Therefore, DMADDM in adhesives could kill the early colonizing bacteria directly and influence the development of biofilm indirectly. DMADDM has been proven to have antibacterial efficacy against eight species of common oral bacteria [43]. However, dental plaque, as a typical biofilm, is a diverse microbial community with a quorum sensing system affecting its formation, development, and resistance to antimicrobials, *etc.* [41]. The initial communities of bacteria found within dental plaque biofilm were of a relatively low diversity in comparison with those present in the mature communities. Co-aggregation bridges existed between these early colonizers then coaggregated with numerous late colonizers. A recent experiment has been designed to investigate the effects of QAMs with different alkyl chain length on three-dimensional biofilms adherent on adhesives for the first time [44]. Further studies, such as an assessment of the detail on multi-species biofilm inhibition, will also be useful.

## 4. Experimental

### 4.1. Synthesis of DMADDM

Dimethylaminododecyl methacrylate (DMADDM) was synthesized via a modified Menschutkin reaction method, which used a tertiary amine group to react with an organo-halide, following previous studies [30]. Briefly, 2-bromoethyl methacrylate (BEMA) was the organo halide, and 1-(dimethylamino)dodecane (DMAD) was the tertiary amine. Ten mmol of DMAD (Tokyo Chemical Industry, Tokyo, Japan) and 10 mmol of BEMA were added in a 20 mL vial with a magnetic stir bar. The vial was capped and stirred at 70 °C for 24 h. After the reaction was complete, the ethanol solvent was removed via evaporation, yielding DMADDM as a clear, colorless, and viscous liquid. Fourier transform infrared (FTIR) spectroscopy (Nicolet 6700, Thermo Scientific, Waltham, MA, USA) spectra of the starting materials and the products were collected between two KBr windows in the 4000 to 400 cm^−1^ region. ^1^H NMR spectra (GSX 270, JEOL, Tokyo, Japan) were taken in deuterated chloroform at a concentration of about 3% [45]. The reactions and the products of DMADDM were verified in preliminary studies [30].

### 4.2. Fabrication of Adhesives Containing DMADDM and Specimen Preparation

Clearfil SE Bond (Kuraray Dental, Tokyo, Japan) was used as the parent bonding system to test the effect of incorporation of antibacterial agents. According to the manufacturer, SE Bond primer contains 2-Hydroxyethyl methacrylate (HEMA), 10-Methacryloyloxydecyl dihydrogen phosphate (MDP), Hydrophilic aliphatic dimethacrylate, dl-Camphorquinone, Water, Accelerators, Dyes and others. SE Bond adhesive contains 2-Hydroxyethyl methacrylate (HEMA), 10-Methacryloyloxydecyl dihydrogen phosphate (MDP), Bisphenol A diglycidylmethacrylate (Bis-GMA), Hydrophobic dimethacrylate, dl-Camphorquinone, *N*,*N*-diethanol-*p*-toluidine, Silanated colloidal silica and others. DMADDM was mixed with SE Bond adhesive, at a DMADDM/(adhesive + DMADDM) mass fraction of 0% (control group), 2.5% and 5%. DMADDM mass fractions of 5.5% or higher were not used due to a decrease in microtensile bond strength in the preliminary study.

The specimens for biofilm experiments were prepared following a previous study [29]. Briefly, composite disks were fabricated using the cover of a sterile 48-well plate (Costar, Corning Inc., Corning, NY, USA) as a mold. Adhesive containing DMADDM or control adhesive was applied over surfaces of composite disks and was light-cured for 10 s. After immersion in distilled water for 24 h to remove unpolymerized monomer, the composite disks with adhesive were sterilized in an ethylene oxide sterilizer (Anprolene AN 74i, Andersen, Haw River, NC, Germany).

### 4.3. Microtensile Bond Strength Test

Microtensile bond strength tests were carried out according to a previous study. Third molars were collected after the donors’ informed consent was obtained under a protocol approved by the West China Hospital of Stomatology, Sichuan University. The roots of the teeth were removed 2–3 mm below the cemento-enamel junction by means of a water-cooled low-speed cutting saw (EMUC6/FC6, Leica Microsystems, Wetzlar, Germany). Then a flat surface was prepared by removing the occlusal one-third of the tooth crowns with the cutting saw to expose midcoronal dentin. The dentin surface was polished with 600-grit SiC paper to create a standardized smear layer [46]. The crown segments were randomly allocated to three groups (*n* = 10), according to the dentin adhesives.

The dentin surface was applied over the primer by using a brush-tipped applicator and rubbed for 15 s. The solvent was removed with a stream of air for 5 s. Then the adhesive was applied and light-cured for 10 s (YOUHONG-1201, YOUHONG Electronics Co., Ltd., Suzhou, China). Resin composite build-ups were constructed with 2-mm increments and light-cured for 60 s.

After storage in deionized water at 37 °C for 24 h, each tooth was vertically sectioned into 0.9-mm-thick serial slabs by means of the a cutting saw with water-cooling. The slabs were sectioned into 0.9 × 0.9 mm composite-dentin beams, excluding those situated peripherally that showed presence of enamel. The beams in each group were immediately subjected to microtensile bond strength testing.

The beams from each group were subjected to tensile testing. Each beam was stressed to failure under tension in computer-controlled Universal Testing Machine (MTS, Eden Prairie, MN, USA) at a cross-head speed of 1 mm/min [46]. The cross-sectional area at the site of failure was measured with a pair of digital calipers (Fisher Scientific, Pittsburg, PA, USA) to calculate the microtensile bond strength values (MPa).

### 4.4. S. mutans Inoculation and Biofilm Formation

The use of *S. mutans* bacteria (ATCC 700610, UA159, American Type Culture, Manassas, VA, USA) was approved by the State Key Laboratory of Oral Diseases, Sichuan University. The growth medium consisted of brain heart infusion (BHI) broth (BD, Franklin Lakes, NJ, USA), which was supplemented with 0.2% sucrose. To prepare the inoculation medium, 15 μL of stock bacteria was added into 15 mL of growth medium and incubated at 37 °C with 5% CO_2_ for 16 h, during which time the *S. mutans* were suspended in the BHI broth. This *S. mutans* culture was then diluted by 10-fold in the growth medium to form the inoculation medium [36].

Each disk was placed in one well of a 24-well plate, inoculated with 1.5 mL of the inoculation medium, and incubated at 5% CO_2_ and 37 °C for 3 days to form mature biofilms [36]. The growth medium was changed every 24 h, by transferring the disks to a new 24-well plate with fresh growth medium. The disks with 4, 24 and 72 h biofilms were used for further analysis.

### 4.5. Lactic Acid Measurement and MTT Assays

The disks with 4, 24 and 72 h biofilms were rinsed in cysteine peptone water (CPW) to remove loose bacteria. Each disk was placed in a new 24-well plate with 1.5 mL of buffered peptone water (BPW) supplemented with 0.2% sucrose. BPW medium was used so that the biofilm would remain stable during the 3 h culture for the acid assay. BPW has a relatively high buffer capacity and the pH would not become significantly acidic, because a low pH would hinder bacterial acid production. Disks with biofilms were incubated at 5% CO_2_ and 37 °C for 3 h to allow the biofilms to produce acid. After 3 h, the BPW solutions were stored for lactate analysis. Lactate concentrations in the BPW solutions were determined using an enzymatic (lactate dehydrogenase) method [47]. A microplate reader (SpectraMax M5) was used to measure the absorbance at 340 nm (optical density OD_340_) for the collected BPW solutions. Standard curves were prepared using a standard lactic acid (Supelco Analytical, Bellefonte, PA, USA).

Then each disk was transferred to a new 24-well plate for the MTT (3-(4,5-Dimethyl-thiazol-2-yl)-2,5-diphenyltetrazolium bromide) assay, which is a colorimetric assay that measures the enzymatic reduction of MTT (a yellow tetrazole) to formazan. This MTT assay for *S. mutans* biofilms was described recently [48]. Briefly, 1 mL of MTT dye (0.5 mg/mL MTT in PBS) was added to each well and incubated at 37 °C in 5% CO_2_ for 1 h. During this process, metabolically active bacteria metabolized the MTT and reduced it to purple formazan inside the living cells. After 1 h, the disks were transferred to a new 24-well plate, 1 mL of dimethyl sulfoxide (DMSO) was added to solubilize the formazan crystals, and the plate was incubated for 20 min with gentle mixing at room temperature in the dark. After brief mixing via pipetting, 200 μL of the DMSO solution from each well was transferred to a 96-well plate, and the absorbance at 540 nm (OD_540_) was measured via the microplate reader. A higher absorbance indicates a higher formazan concentration, which in turn indicates more metabolic activity in the biofilm present on the composite disk.

### 4.6. Exopolysaccharides (EPS) Staining

Exopolysaccharides (EPS) assay was conducted according to previous studies [37]. Briefly, the bacterial cells were labeled by using 2.5 μM SYTO 9 green fluorescent nucleic acid stain (Molecular Probes, Invitrogen Corp., Carlsbad, CA, USA). The polysaccharides were labeled with 2.5 μM Alexa Fluor 647-dextran conjugate (Molecular Probes, Invitrogen Corp., Carlsbad, CA, USA). Then disks with 4, 24 and 72 h biofilms were examined using confocal laser scanning microscopy (CLSM) (Leica, Wetzlar, Germany).

Partly quantified analysis of EPS production and bacteria was achieved with Image-Pro Plus 6.0 (Media Cybernetics, Inc., Silver Spring, MD, USA) by calculating the value of relative fluorescence (the bacteria stained by SYTO 9: green; the EPS stained by Alexa Fluor 647: red).

### 4.7. Live-Dead Bacteria Staining

After 4, 24 and 72 h, the biofilms on the disks were washed three times with PBS, and then stained using the BacLight live/dead bacterial viability kit (Molecular Probes, Eugene, OR, USA). Live bacteria were stained with Syto 9 to produce green fluorescence, and bacteria with compromised membranes were stained with propidium iodide to produce a red fluorescence. Disks were examined using a confocal laser scanning microscopy (CLSM) (Leica, Wetzlar, Germany).

### 4.8. Scanning Electron Microscopy

Biofilms on the disks at 4, 24 and 72 h were rinsed with PBS and then immersed in 1% glutaraldehyde in PBS for 4 h at 4 °C. The specimens were rinsed with PBS, subjected to graded ethanol dehydrations, and rinsed twice with 100% hexamethyldisilazane. The specimens were then sputter-coated with gold and examined via scanning electron microscopy (SEM, Quanta 200, FEI, Hillsboro, OR, USA).

### 4.9. Statistical Analysis

One-way analysis of variance (ANOVA) was performed to detect the significant effects of the variables. Tukey’s multiple comparison test was used to compare the data at a *p*-value of 0.05. Each standard deviation (SD) serves as the estimate for the standard uncertainty associated with a particular measurement.

## 5. Conclusions

The adhesives containing DMADDM inhibited *S.* *mutans* biofilm growth, lactic acid production and EPS metabolism. The novel adhesives had inhibitory effects on both bacteria and matrix in *S. mutans* biofilms at different developmental stages. Lower mass fractions of DMADDM had similar antibacterial effects as higher mass fractions of DMADDM on initial biofilms. However, adhesives with 5% DMADDM showed stronger anti-biofilm potential on the mature biofilm than the adhesives with 2.5% DMADDM. Further, the dentin adhesive bond strength of the novel antibacterial adhesives in the present study matched that of the control commercial product. The new adhesives investigated in the present study therefore show promise for the control of dental plaque and the reduction of secondary caries.
